# Understanding communication pathways to foster community engagement for health improvement in North West Pakistan

**DOI:** 10.1186/s12889-016-3222-7

**Published:** 2016-07-18

**Authors:** Monique Lhussier, Nicola Lowe, Elizabeth Westaway, Fiona Dykes, Mick McKeown, Akhtar Munir, Saba Tahir, Mukhtiar Zaman

**Affiliations:** Faculty of Health and Life Sciences, Northumbria University Coach Lane Campus East (H005), Longbenton, Newcastle upon Tyne, NE7 7XA UK; International Institute of Nutritional Sciences and Applied Food Safety Studies, School of Sport and Wellbeing, University of Central Lancashire, Preston, UK; Maternal and Infant Nutrition and Nurture Unit (MAINN), School of Community Health and Midwifery, University of Central Lancashire, Preston, UK; School of Nursing, University of Central Lancashire, Preston, UK; Abaseen Foundation, Peshawar, Khyber Pakhtunkhwa Pakistan; Khyber Medical University, Khyber Teaching Hospital, Peshawar, Khyber Pakhtunkhwa Pakistan

**Keywords:** Community engagement, Iodised salt, Communication, Influence, Health improvement

## Abstract

**Background:**

This paper describes the community engagement process undertaken to ascertain the focus, development and implementation of an intervention to improve iodised salt consumption in rural communities in North West Pakistan. The *Jirga* is a traditional informal structure, which gathers men respected within their community and acts in a governing and decision-making capacity in the *Pukhtoon* culture. The *Jirga* system had a dual purpose for the study: to access men from the community to discuss the importance of iodised salt, and as an engagement process for the intervention.

**Methods:**

A number of qualitative data collection activities were undertaken, with *Jirga* members and their wives, male and female outreach workers and two groups of women, under and over 40 years old. The aim of these was to highlight the communication channels and levers of influence on health behaviour, which were multiple and complex and all needed to be taken into consideration in order to ensure successful and locally sensitive community engagement.

**Results:**

Communication channels are described within local families and the communities around them. The key influential role of the *Jirga* is highlighted as linked both to the standing of its members and the community cohesion ethos that it embodies. Engaging *Jirga* members in discussions about iodised salt was key in designing an intervention that would activate the most influential levers to decision making in the community. Gendered decision-making processes within the household have been highlighted as restricting women’s autonomy. Whilst in one respect our data confirm this, a more complex hierarchy of decisional power has been highlighted, whereby the concept of ‘wisdom’- an amalgamation of age, experience and education- presents important possibilities. Community members with the least autonomy are the youngest uneducated females, who rely on a web of socially and culturally determined ways to influence decision-making.

**Conclusions:**

The major lines of communication and influence in the local community described are placed within the wider literature on community engagement in health improvement. The process of maximisation of local cultural knowledge as part of a community engagement effort is one that has application well beyond the particular setting of this study.

## Background

This study was undertaken in rural communities on the outskirts of Peshawar in North West Pakistan. Many community members live and work on brick kilns, and have an average income of less than one US dollar a day. The mixed population of local Pakistanis and long-term Afghan refugees are mostly from the *Pukhtoon* ethnic group, with a traditionally tribal and patriarchal culture. This Wellcome Trust funded project was conducted as part of a long-term collaboration between the Abaseen Foundation Pakistan (AFPK), a locally operating Non-Governmental Organisation, and UK-based universities for a number of years to improve health, wellbeing and education in Pakistan [1–3].

In Pakistan, very substantial differences persist between regions and different socio-economic groups in access to resources for health improvement [4]. A small percentage of educated and/or wealthy Pakistani families have access to healthcare and health promotion information, but many do not [5]. Many people live below the poverty line, in unsanitary conditions with limited access to clean water, are poorly educated and have unequal access to education and healthcare [5, 6]. The project reported here focussed on the role of the *Jirga* in engaging the community in health improvement, despite these challenging contextual conditions. The *Jirga* is a traditional informal structure, which gathers men respected within their community and acts in a governing and decision-making capacity in the *Pukhtoon* culture [7, 8].

In spite of the launch of Pakistan’s national Iodine Deficiency Disorders Control Programme in 1994, approximately half of Pakistan’s population of 200 million are affected with Iodine Deficiency Disorders [9]. A National Nutrition Survey revealed marked provincial variation; with for example 25.7 % of children aged 6–12 years at risk of iodine deficiency in the north western province where this project took place [10]. To build on this, we conducted a baseline household survey in May–July 2012, including 1,043 local households. It showed in particular that 97 % of households did not use iodised salt and reported not to understand its benefits. A *Jirga* was called to share the findings and discuss what could be addressed within the timescale and funding parameters of this project. This led to the development of a multicomponent awareness raising campaign, promoting the benefits of iodised salt to improve knowledge, attitudes and practices in the area.

### The overall project

This project involved a team of researchers in the UK (NL, EW, MMcK, FD & ML), a research lead in Pakistan (MZ) and Pakistan-based research assistants (AM & ST), and the Abaseen Foundation. All UK-based researchers have Doctorates and a combined wealth of experience in qualitative and quantitative research methodologies, and the lead PK researcher has an MD, and a long term engagement with the team. With such a widespread geographical area and broad expertise, regular communication loops were key to the success of the project, in particular to ensure that the model was not directed by ethnocentric conceptualisations and understandings of community engagement, but led by the Pakistan-based team. This was operationalised as a two-way communication loop, with weekly Skype meetings being held between the Pakistan and UK-based researchers and additional Skype training sessions being offered by the UK team in qualitative methodologies.

The following steps were used in the design, implementation and evaluation of the intervention:

Step 1: Focus Group Discussions (FGDs) were conducted with community members to gain a greater understanding of the knowledge related to iodised salt, its intake and local availability, and the role of different stakeholders in promoting its use.
Step 2: The intervention, a multi-component community awareness raising campaign, was implemented over a 4-month period from June to September 2013.
Step 3: A survey of local shopkeepers was undertaken prior to the start of the intervention and during the intervention to collect data about the types and quantity of salt sold, its storage, packaging and pricing. Further monitoring of sales was conducted on a monthly basis.
Step 4: The intervention was evaluated:
  ○ Quantitatively, based on salt sales figures (pre and post-intervention) and through the Urinary Iodine Concentration (UIC) of school boys aged 6–12 years taken at three time points [11].
  ○ Qualitatively, using a post-intervention semi-structured questionnaire to collect data on knowledge, attitudes and practices towards iodised salt and its use.

The evaluation has been published separately [11]. Iodised salt sales and UIC were monitored to assess the effectiveness of the intervention. At baseline, 2.6 % of households reported use of iodised salt. During the intervention, sales of salt labelled as iodised increased by 45 %, however this was not reflected in an increased UIC. Therefore, whilst the intervention was successful in terms of raising awareness and changing behaviours, issues remain regarding adequate iodisation by local producers and appropriate storage of salt.

This paper describes the community engagement process undertaken to ascertain the focus, development and implementation of the intervention, situating this in the broader literature on community engagement in health improvement. Iodised salt is thus used here as a case example for future engagement and health improvement interventions in culturally specific settings. The paper adheres to the COREQ guidance on reporting.

### Community engagement and health improvement

The fact that community participation is a key factor in successful health promotion initiatives has long been acknowledged internationally [12–14]. Broadly speaking, models of engagement have been conceptualised along a hierarchy of levels, ranging from non-participation (at the bottom of the hierarchy), information giving, through to consultation, joint decision-making, and full community control or self - mobilisation (at the top) [15–17]. Whilst the difficulty of evaluating the impact of community engagement initiatives has been widely acknowledged because of the great variety of approaches and settings [15, 18], there is now solid evidence that they have a positive impact on a range of health outcomes, such as health behaviours, health consequences, participant self-efficacy, and participant social support [19, 20]. A recent meta-analysis of community engagement highlighted that interventions that engaged the community in delivery as those with the largest pooled effect size [19]. Change is then believed to be facilitated by the credibility, expertise or empathy that the lay community member(s) can bring to the intervention delivery [21]. Whilst it has been acknowledged that many interventions use combinations of engagement methods [15], much of the research undertaken to−date has focused on developed countries. This article responds to calls to document with greater clarity community participation strategies used in health promotion research in order to maximise translation potential to other contexts [4, 14]. We focus on a particular socio−cultural context, in North West Pakistan, and describe the relationships between this context and the possibilities and constraints related to community engagement work.

The *Pukhtoon Jirga* system has been defined as a gathering of all men concerned with a specific issue, plus others who are respected or influential locally [22]. Representation in the *Jirga* is based on alliances, lineage, patronage and/or cultural value orientations, and is thus fluid and topic-specific. Without the backing of community leaders and representatives through this system, it would have been impossible to conduct the research. However, communication channels and levers of influence on health behaviour, like in many other societies, are multiple and complex and all needed to be taken into consideration in order to ensure successful and locally sensitive community engagement. The *Jirga* system had a dual purpose for the study; to access men from the community and discuss the importance of iodised salt, and as an engagement process for the intervention.

## Objectives

The objective of the study was to explore the naturally occurring communication and influence channels in the local communities to ensure effective engagement in a programme to promote increased use of iodised salt.

## Methods

Drawing on principles of ethnography, qualitative data were collected as part of step 1 described above, focussing on 1) the *Jirga*, their members and immediate networks, and 2) broader community groups. Unless otherwise stated, the sampling strategy was purposeful, drawing on the web of relationships MZ and AFPK already had in the community (we return to this point in the discussion), and therefore we had no case of refusal to participate. Interview guides were developed collaboratively between the UK and Pakistan-based teams, and tailored to the participant(s).

### Activity 1-*Jirga* members

Over a period of 15 months from March 2012 to June 2013, AFPK called seven *Jirgas* to discuss operational activities related to community development. *Jirga* members were selected on the basis of their experience and reputation, and invited to participate by the male research assistant to attend both verbally by visiting their homes or work place one week before and also by reminding them by mobile phone the day before the *Jirga* took place. Apart from one, all *Jirgas* were audiotaped after obtaining consent from the participants, and they lasted approximately 90 min. Proceedings of the *Jirgas* were facilitated by MZ. Recordings were transcribed and translated into English, anonymised and analysed using NVivo 10.

In addition, one-to-one interviews with four *Jirga* members, and a focus group with nine *Jirga* members were conducted in the local community hospital (lasting approximately 50 min) to gain a greater understanding of the *Jirga* system and its role in community development, particularly in relation to health improvement. For the interviews, a list was compiled, with the assistance of local school teachers of sixteen men who were active members of *Jirga* within a 5 km radius of the local Health Centre. A random sample of four *Jirga* members was selected; they were invited to take part and all agreed. Three interviews took place in the local *Hujra* (venue for social gatherings), and one at the participant’s home; they lasted 30 min on average. For the focus group, participants were recruited using a snowballing sampling method from one AFPK member.

Semi-structured interviews were also undertaken with the wives of *Jirga* members to investigate if and how *Jirga* discussions were shared with the community. Four *Jirga* members (different from above) were approached to seek their permission for the female Pakistan-based research assistant to interview their wives. The four households were visited, wherein the purpose of the study was explained, consent obtained and semi-structured interviews conducted. A total of eight women were interviewed from these four households (two per house), whose ages ranged from 35 to 70 years. In order to protect their anonymity, no direct quotes are reported in this paper, but rather the researcher’s notes following a discussion.

### Activity 2-community groups

FGDs were conducted with different community groups to gain a greater understanding of the role of *Jirga* members in promoting health locally. These included:

1. Women under 40 years old;
2. Women over 40 years old;
3. Lady Health Workers (female outreach workers);
4. Male informal healthcare workers.

The rationale for selecting two distinct groups of women was based on the premise that older women (e.g. mothers-in-law) often make healthcare decisions for the family. Sampling was purposeful. Four FGDs were thus conducted, each lasting approximately 40 min. Due to cultural constraints, the female research assistant facilitated three FGDs with female participants and the male research assistant facilitated one FGD with male participants.

FGDs were held with nine women over 40 years old and eight women under 40 years old in November 2012. Two further FGDs involving seven female outreach workers and eight male informal healthcare workers were conducted in December 2012 and March 2013, respectively.

### Analytical strategy

Apart from one *Jirga* and one face-to-face interview where consent to record was declined, all interviews and focus groups were audio-recorded, and additional field notes were taken. Recordings were transcribed and translated into English, anonymised and thematically analysed using NVivo 10. This involves coding the data to extract basic themes; these are then collated into organising and finally global themes [23]. These included agency, communication, Jirga membership, poverty, power and hierarchy, religion and traditions. Data was coded and analysed inductively by both the Pakistan and UK-based research teams, which included a triangulation of findings between the different participating groups. This was a highly iterative process, whereby the comprehensiveness of the data was thus ensured, and any gap in knowledge addressed through discussion with the Pakistan-based team. The transcripts were not returned to research participants, as this research was not about eliciting individual, but communal experiences, and the Pakistan-based team ensured the validity of all of the cultural understandings developed from the data and reported here.

## Results

It was important for the research team to develop a thorough understanding of the local channels of communication and influence to understand how to communicate information in the most effective way. A number of themes emerged from the data, pertaining to the decision-making processes in the family, communication channels in the community, and then more precisely in and around the *Jirga*. These are detailed in turn below.

### Decision-making in the family environment

**Fig. 1:**
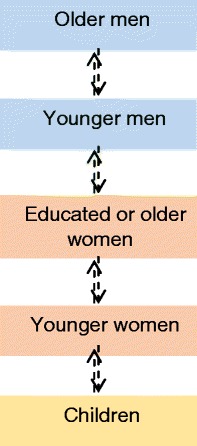
Lines of communication within local families

Throughout the project, the Pakistan-based researchers provided regular clarifications and notes of cultural sensitisation for the UK-based team. They highlighted the following family hierarchy: if the male head of family is alive, he has the power to make all family related decisions without consulting any family member. The older he is, the wiser he is considered to be and the less likely it is that his decisions are disputed. Where the head of family has passed away or is absent, that role is passed on to the oldest woman of the household, who will usually make decisions in collaboration with her eldest son. If the son is old enough to be married and considered mature, then he takes on the role of head of family. This is represented in Fig. 1.

Community members are grouped by gender and placed in order of decreasing decisional power. Younger women explained this further in relation to grocery shopping and the local customary restrictions over their movements:

*Who brings groceries for you?*

*Men.*

*So when men are out of homes, then what do you do?*

*Then we ask the children or men of the village to go to the market and bring us groceries or whatever it is that we need.*

*So when men and children both are not around, then how do you manage to buy groceries from the market?*

*Then we ask from one another to share with us their groceries, women of this area do not go out to buy groceries.* (FGD with women under 40).

Whilst physically going to the market was the remit of men and boys, the decision as to what had to be purchased varied somewhat:

*… In rural areas people are not educated, so the decision is made by men of the house. Educated women just hand over a list of required items to their male counterparts to buy and bring the required items.* (FGD with men Informal Healthcare Workers)

Younger women agreed that on the whole locally, men were key decision makers when it came to grocery shopping:

*Men bring whatever they like, we can’t ask anything about whether you should bring iodine salt or simple salt, and we just use whatever he brings home*.... *if women complain about the type of groceries the men bring in, they feel offended and threaten to not bring it all.* (FGD with women under 40)

This was corroborated by participating men:

*Obviously the male member should go and also decide the type of salt to buy… As he has to pay for it. … The women are very simple village type so whatever we bring home they use it.* (*Jirga* FGD)

This key decision-making role meant that we ensured men were engaged in our iodised salt awareness raising activities. A number of traditional beliefs emerged through the data, which reinforced the need for us to engage the most influential community members in the intervention. These linked goitres to throat infections, depression or the consumption of unclean water, and a fear that iodised salt may lead to birth control. Older women highlighted how the lines of communication between genders, although bound by clear traditional rules, are not always straightforward and there are clear exceptions:

*The normal salt is wrapped in a smaller packet which is cheap whereas the iodised salt is in a big packet. My wife … asks me to bring the big packet even though the small packet is cheaper.* (*Jirga* FGD)

Older women in particular could clearly be influential in family decision making:

*My grandmother once had goitre, so now whenever we need salt in the house, she tells us to bring iodised salt only… She knows the importance of iodine salt and the problems connected with the deficiency of iodine.* (FGD with Male Informal Healthcare Workers)

On domestic issues, mothers-in-law hold considerable decisional powers, as was described in one of the *Jirgas*:

*A lady, who had some health problem … came to our home with her mother-in-law for spiritual treatment.... I told her that Prophet said that … If a person recites chapter Fatiha seven times on the ill person … so God will heal him… I told that mother-in-law that besides doing this, take her to doctor as well. So I told her that you dance in your son’s wedding and beat the drum, so then take this unfortunate to a doctor, who is someone else’s daughter, so that she gets fine. So she replied me… God will heal her and that mother-in-law just wants not to spend the money of her son…* (*Jirga* meeting)

Two key points emerge from this quote; the fact that mothers-in-law make decisions when it comes to the health of younger brides, and the fact that in the community older women do communicate with, and question older, wiser men.

### Communication channels in the community

We sought to understand the most effective communication channels beyond the family, in relation to health improvement. Men hold regular informal meetings:

*We sit in our Hujra every night, almost 20 to 30 people… sometimes we hear a case of a child having iodine deficiency and I tell them that it’s because of iodine. I tell them but they don’t listen.* (FGD with Male Informal Healthcare workers)

Women sometimes can communicate with the community at large through the male members of their family.

*Well my 13-year-old son goes to mosque, you can write me down the message and I will give it to him to post it in the mosque or distribute it to everyone. I am not educated but others are. We can spread this message in the house of ALLAH. It’s a very right thing to do*. (FGD with women over 40)

Religious leaders are highly regarded in all matters, including health:

*If Ulema [religious leader] say something like this [iodised salt], everyone listens to him because he is very influential*. (FGD with women under 40)

Women under 40 years old, who traditionally have very little decisional or influential powers, suggested ways in which a health message about using iodised salt may be spread effectively:

*We can inform our neighbours, relatives, whoever we meet, about the uses and benefits of iodine salt.*

*We will also inform our men who can also spread the message amongst their friends, relatives, people in mosque and Hujra.*

*Teachers can inform children about the use of iodine salt who can then pass the information to their parents…* (FGD with women under 40)

Thus, it appears that younger women may not solely be the passive recipients of others’ decisions, but gladly offer to spread the message among their peers. Women can talk to women of their age and standing, and occasionally speak to men who can then spread the message through their male networks. Education and influence on children is also clearly valued. Other channels were suggested:

*There is not much TV here, but everyone has a radio and it would be very helpful if they run it on radio.*

*[Doctors] can inform all the women who come and visit the hospital.* (FGD with women under 40)

Whilst the radio was a valued communication means for women who might spend extensive amounts of time at home, men favoured word of mouth:

*We do not listen to radio but mostly talk among ourselves enjoying warmth of sun.* (*Jirga* FGD)

Men made similar suggestions for the effective spread of a health education message, emphasising the use of influential and respected community figures, though not clarifying how the message would then reach women:

*Like the Imam of Mosque may talk during Friday sermons … regarding the use of iodised salt and its positive effects on the health of children and the whole household. The school teacher can tell the students in a formal lesson and highlight the benefits and also the problems caused if not used. In the same way the Hujra elders can inform the younger people.* (*Jirga* FGD)

In cases where the gravity of the situation calls for it, or when male relatives are absent, women are able to talk to older, wise men in order to resolve an issue:

*My wife listens to the female when she comes personally to the Jirga…. Sometimes I listen to the female… These people are Afridi by tribe and by profession they are drivers… they come to home after one month. This lady stopped me in the middle of the road… and said that [someone] has forfeited her property by force and transferred [it] to this name. … I … reached to those people to whom the woman accused. Those people were very influential. One of them was refusing. But I took the authority and declared the final decision. After a week her husband came to me and thanked me a lot to have finalised the matter in his absence*. (Interview with JM-01-H)

**Fig. 2:**
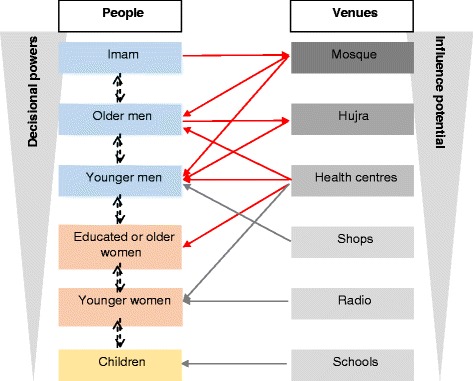
Lines of communication in the local communities

It transpires from this data that lines of communications do not solely depend on a person’s standing within the family or community, but particular venues can form key communication vehicles too. This is represented in Fig. 2:

Venues are colour coded so that the darker ones exert the greatest influence on attendees. Red arrows show lines of influence: Imams influence people’s behaviours through their communications at the Mosque (in the actual Mosque and through loudspeakers for the benefit of the whole community), which is mostly attended by men, young (including boys) and old. Men also gather at the *Hujra*, where wiser men counsel younger ones.

The grey arrows indicate lines of communication through which information is disseminated. Male health care professionals working in health centres have influence on adult men and may impact on health related decision-making in the family. They may also disseminate information on improving health behaviours to women patients.

Shop owners, radio stations and school teachers mostly have a role in the dissemination of a health promoting message. Communication also happens directly through the ranks of decisional powers (from children, particularly boys, to older men), often echelon by echelon, but not always, as the data shows. The levers for community engagement are therefore linked to social influence through gender, age, religion, wealth and education.

FGD data was crucial for understanding barriers to the use of iodised salt, and helped in designing an intervention that would activate the key levers to decision-making in the community. This resulted in a multi-component awareness raising campaign that included: the design and distribution of leaflets and posters at the Mosque, *Hujra*, Health Centres and schools, to shopkeepers and informal healthcare providers, and to households, brick kilns and communal areas. Health education sessions were also given to groups (*Jirga* members, healthcare providers, school teachers) and individuals (door-to-door visits). In addition, a radio interview with the Pakistan-based project lead (MZ) was broadcast on the local radio station. Such comprehensive approaches to information dissemination and social mobilisation is in line with recommendations found in the literature [4].

### Communication within and around *Jirgas*

Since the *Jirga* system was a prominent part of the local communities, it was important to understand modes of communication within and around it. In their field notes, the Pakistan-based research assistants noted the working of the *Jirga*:

*Before the Jirga each member meets each other… in a traditional way by shaking hands and hugging each other in a smiling mood and also asking about each other’s life that how is life going on asking about each other family members and work…. The Jirga was started with the recitation of the Holy Quran after that all the event was gone in a very light mood all the members give respect to each other and listened to each other very carefully* … (*Jirga* Meeting Observation Report)

Focus group members described the function and status of the *Jirga* as a gathering of elder or respected community members, who often meet to resolve local conflicts:

*If someone has any problem, then elders of the locality get together to find out the solution of the matter. The right solution.* (FGD with Lady Health Workers)

The respect inspired by decisions taken at *Jirga* level was very apparent in the data. Further explanation is given below about the community function of such resolution:

*If for example someone kills anyone and his elders go to court and police captures the accused, role of the court is to give punishment to a prisoner, still the system doesn’t turn their dispute into friendship. So this is the difference. Jirga tells them to live friendly and brotherly*. (Interview with JM-02)

JM-02 qualified this with an example of the *Jirga*’s emphasis on community cohesion:

*Once my brother had a clash with someone … [they] had fight and my brother shoot few people of other party. When I came to know about this clash I ran quickly and took all the injured people to the hospital for treatment before knowing and reaching the family members of the other party, my brother was killed… After the death of my younger brother… the elders of the village gathered at one place and we did the Jirga. Jirga decided that I will give him two hundred thousand rupees and will arrange a dinner for the whole Jirga along with two sheep. On the day of Jirga I took all the things but other party returned other cash and took only one sheep and arranged the dinner personally for the entire Jirga, thus our clash turned into friendship. Till today we are living happily. My nephews sit together with them.* (Interview with JM-02)

Participants described how the function of *Jirgas* had evolved from mostly being about conflict resolution to including a greater emphasis on social aspects of community life:

*The social work of the village is [a] recent development of the Jirga. It was not so in the past… There are numerous problems like health […] and developmental works cannot be overcome without the efforts of these elders*. (*Jirga* FGD)

With changing times, there was therefore an increased potential that the *Jirga* system might engage in local health issues, thus taking on functions beyond dispute resolution. Participants described *Jirga* membership as flexible, depending on the issue at hand. As a rule, membership was aligned with status and wisdom:

*The ‘white bearded’; they are known people whom the people ask for Jirgas. People know well and he is a well-known person and he is the one who attends every Jirga. People invite him to come for solving their problems*. (FGD women over 40)

Whilst age is generally respected, this does not preclude younger men from being invited to a *Jirga*. Wisdom and ‘strength’ are recognised and valued:

*Even a younger person can be the Jirga elder … Some of the men are strong [wise]…. Even some women are strong and others are weak like me…. In our locality this woman is strong enough to talk issues with men…* (FGD women over 40)

This highlights the potential for community engagement through the *Jirga* around health issues, whereby behaviour change needs to happen at household level. Nevertheless, the status that comes with wealth and education are key factors in membership:

*They look somehow for either the malik [religious leader], or white bearded or look for a rich person. Nobody brings a poor man. … the poor is looked down upon*. (FGD women under 40)

Decisions are mediated, formalised and invariably respected, denoting the influential impact of *Jirgas* and the respect they command. This is just as salient for *Jirgas* that focus on health maintenance and disease prevention, for example in relation to sanitation:

*Villagers get assembled and it is conveyed to them to … dig streams for getting new water, clean the water channels and the dirt is (taken away)…. In our area it is such like that the residents of […] get together and collect Rs. 100 or Rs. 150 from each home. Then the money is given to a worker who cleans the drain and throws away the dirt …. The elders of [the area] get together about the sanitation [management].* (FGD with Lady Health Workers)

*Jirga* members’ wives agreed with this general acknowledgement of respected influence and its potential to improve health behaviours:

*All the females are of the opinion that the Jirga can play significant role in health because the Jirga members have strong position in the community; they can mobilise the people for cleanliness campaign(s) … they can also utilise community volunteers to create any type of effective awareness.* (*Jirga* Members Wives discussion notes)

**Fig. 3:**
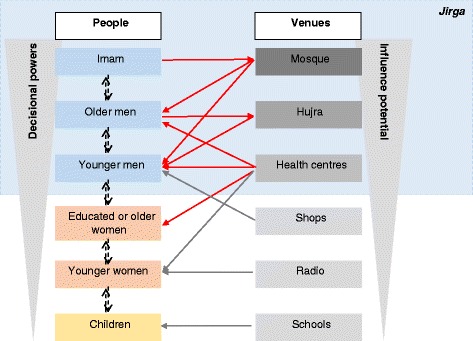
Lines of communication and influence in the local communities, mediated through the *Jirga* system

What emerged is that the most likely effective engagement channels are those that are built on levers of age, gender and education – i.e. the *Jirga* works because it gathers men who are respected in the community on the basis of their status related to age, wealth, ‘wisdom’ and education level. Because of the respect commanded by *Jirga* decisions, it became clear that only an intervention engaging *Jirga* members would challenge deeply embedded cultural misconceptions about iodised salt. Figure 3 highlights the prominence of the *Jirga* in the local communities.

## Discussion

This research focussed on communication channels in local families, the communities around them, and highlighted the key influential role of the *Jirga*, linked both to the standing of its members and the community cohesion ethos that it embodies. Gendered decision making-processes within the household have been highlighted as restricting women’s autonomy [24]. Whilst in one respect our data confirm this, a more complex hierarchy of decisional power has been highlighted, whereby the concept of ‘wisdom’, an amalgamation of age, experience and education, presents important possibilities. Community members with the least autonomy are therefore the youngest uneducated females, who rely on a web of socially and culturally determined ways to influence decision-making. Engaging *Jirga* members in discussions about iodised salt was key in understanding barriers to use, and designing an intervention that would activate the most influential levers to decision making in the community. This resulted in a multi-component awareness raising campaign that included: the design and distribution of leaflets and posters in key venues; the delivery of health education sessions to groups (*Jirga* members, healthcare providers, school teachers) and individuals (door to door visits), as well as the broadcast of an interview on local radio. Such a variety of engagement strategies attuned to the local community characteristics maximised the effectiveness of our intervention, despite challenging structural conditions.

Elmusharaf et al. [24] highlight the limitations of strategies to improve the demand for health services in resource-limited settings. These include a lack of knowledge of the target community, resulting in a lack of understanding of three fundamental influences on the decision-making environment: “gendered decision making norms, multigenerational dialogue and appropriate communication.” To date, the literature on the *Jirga* has been descriptive, failing to generate in-depth understandings about how and why the model works [7, 8, 22]. Within this particular socio-cultural context, the *Jirga* exemplifies elements of what we would recognise as deliberative democracy and addresses some of the three limitations above [24]. Relatively few models of community engagement have sought to capitalise on such existing and culturally embedded mechanisms of communication and influence. This paper sought to bring together these two research strands (Jirga and community engagement) in order to develop a bespoke community engagement strategy around the use of iodised salt

Community engagement can, of course, vary during the lifetime of a project. Snijder et al. [14] have recently developed a matrix of community engagement across the four stages of project development: diagnosis of the issue, development of appropriate strategies, implementation of the intervention and evaluation. For each stage, they detail seven levels of community participation: non-participation; passive participation; participation by information; participation by consultation; functional participation; interactive participation and self-mobilisation. Their study builds in part on O’Mara-Eves et al.’s [19] research, which compares the effectiveness of four models of community engagement:

1. When the health need is identified by the community and they mobilise themselves into action;
2. Where the need for an intervention is driven from outside of the community, but community insiders are consulted over its development;
3. Where the need for an intervention is driven from outside of the community, but community insiders collaborate over its development;
4. Where the community is involved in the delivery of the intervention only.

O’Mara-Eves et al. [19] found the fourth mode of community engagement to be particularly effective and postulated that this might be linked to the expertise and credibility of empathy of the intervention deliverers.

Our intervention adds to this framework by arguing that a sound understanding of community dynamics, lines of influence and communication can only help tailor a community engagement approach. Hence, we engaged with the *Jirga* first and foremost, in order to determine the focus of the intervention. This was a combination of models: 1 (the initial survey established areas of need), 2 (iodised salt consumption was chosen as a topic because it was achievable in the timescale and funding limitations of this project) and 3 (*Jirga* and other community members advised on the most effective lines of communication). Maximising the potential of these influential entry points into the community was key in ensuring the intervention success. Once the focus of the intervention was decided, groups were consulted over the best ways to ensure effectiveness (model 2), and local outreach workers delivered some of the intervention, as did Imams, health care workers and shop keepers (model 4). Beyond this, this intervention also built on long standing and trusting relationships between AFPK workers and the local community.

### Strengths and weaknesses

This research took part in a relatively small area, in North West Pakistan and the extent to which our findings can be translated to other parts of the world needs to be considered carefully. The process undertaken does, however, have relevance for many other settings. Explicitly exploring naturally occurring channels of communication of influence in any community prior to setting up a health improvement intervention should help maximising its effectiveness potential.

## Conclusion

This article provides an example of an intervention developed in a particularly challenging cultural and socio-economic context, in North West Pakistan. A variety of engagement strategies attuned to the local community characteristics maximised the effectiveness of our intervention, despite challenging structural conditions. Whilst gendered decision-making processes are well acknowledged, and in one respect our data confirm this, a more complex hierarchy of decisional power has been highlighted. The concept of ‘wisdom’, an amalgamation of age, experience and education, presents important possibilities that should be maximised in future health improvement efforts. Referring back to Snijder et al’s [14] matrix, partly for pragmatic reasons linked to the distance between the UK and Pakistan-based teams, which limited the control that the UK-based team could exert, and partly due to our moral intent, the driving modus operandum throughout the project was one of interactive participation. This process of maximisation of local cultural knowledge prior to, and as part of, a community engagement effort in order to understand the levers of influence and communication is one that has application well beyond the particular setting of our study.

## Abbreviations

AFPK, Abaseen Foundation Pakistan; FGDs, focus group discussions; UIC, urinary iodine concentration; UK, United Kingdom
